# Aortic arch width by CMR is highly reproducible between readers and across imaging sequences

**DOI:** 10.1186/1532-429X-18-S1-P143

**Published:** 2016-01-27

**Authors:** Saadia Qazi, Christopher J O'Donnell, Warren J Manning, Michael L Chuang

**Affiliations:** 1 NHLBI's Framingham Heart Study, Framingham, MA USA; 2 grid.239395.70000000090118547Cardiology, Beth Israel Deaconess Medical Center, Boston, MA USA; 3 Cardiology, VA Healthcare System, Boston, MA USA; 4 grid.416488.7Internal Medicine, North Shore Medical Center, Salem, MA USA

## Background

Aortic arch width (AAW) is defined as the distance between the centroids of the ascending and descending thoracic aorta at the level of the main pulmonary artery bifurcation, measured from an axial image. Previously, in a cohort of >3000 community-dwelling adults who underwent chest computed tomography for research purposes, we found that increased AAW is an independent predictor of incident adverse cardiovascular disease (CVD) events, even after adjustment for traditional CVD risk factors, and for coronary artery calcium. Thus AAW may be useful for risk stratification. In this study, we sought to assess the agreement in AAW measured using two CMR sequences: black-blood (BB) and phase contrast flow (QF) methods. We hypothesized that AAW would not differ between the two sequences, and that reader reproducibility would be high regardless of sequence.

## Methods

A single reader measured AAW in 50 consecutive patients who underwent clinically-indicated CMR that included T1W TSE BB (211x512 matrix; 320-mm FOV; 5-mm THK) and QF (128x288; 320-mm FOV; 6-mm THK) sequences. For each sequence AAW was measured at the level of pulmonary artery bifurcation, or if that was not visualized, at the right pulmonary artery level; QF measurements were made from the magnitude image. All scanning was performed on a 1.5T system with patients supine. Thirty of the 50 patients were analyzed by a second reader unaware of the primary reader's results; the primary reader re-analyzed the same 30 cases two weeks after initial analyses and blinded to prior results. Agreement between sequences was assessed using the Bland-Altman technique; for completeness we also compared results by Pearson correlation and paired t test. Observer reproducibility was assessed using intraclass correlation coefficient (ICC). Results are summarized as mean ± standard deviation.

## Results

The 50 patients (18 women) were aged 54 ± 16 years [range 19, 85] with body surface area=1.94 ± 0.27m^2^ [range 1.49, 2.48]. BB AAW=78.1 ± 12.3 mm [range 49.2, 100.9] did not differ from QF AAW=78.2 ± 12.0 mm [range 50.6, 100.4] by paired t test, p = 0.40; Pearson correlation was r = 0.99. Bland-Altman analyses showed excellent agreement, mean bias=-0.15mm, 2SD=2.52 mm (Figure: solid line=mean bias, dashed lines=95% confidence intervals). Interobserver ICC=0.990 for BB and 0.994 for QF; intraobserver ICC=0.991 (BB) and 0.992 (QF).

## Conclusions

AAW measured from black-blood CMR images did not differ from AAW determined using (bright-blood magnitude) images from phase-contrast flow CMR, despite diffrerences in spatial resolution and vessel contrast. Intersequence correlation was high and observer reproducibility was excellent for both sequences. These results suggest that AAW can be determined reliably using a variety of CMR sequences, and that the AAWs so obtained can be meaningfully compared with each other (e.g. across serial studies).

**Figure 1:**
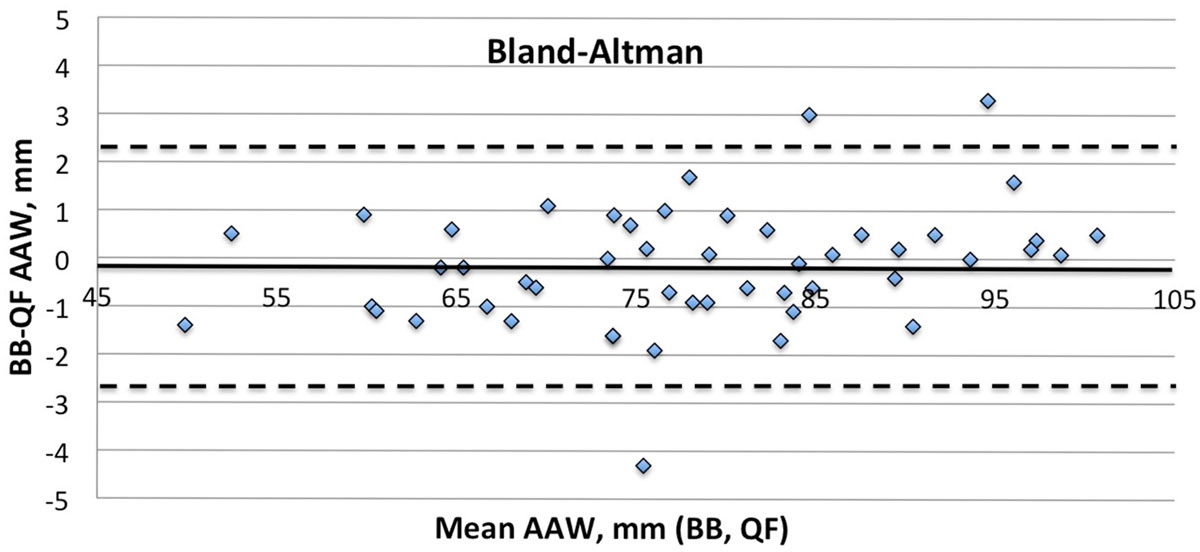
Figure 1

